# A possible link between coral reef success, crustose coralline algae and the evolution of herbivory

**DOI:** 10.1038/s41598-020-73900-9

**Published:** 2020-10-20

**Authors:** Sebastian Teichert, Manuel Steinbauer, Wolfgang Kiessling

**Affiliations:** 1 Fachgruppe Paläoumwelt, GeoZentrum Nordbayern, Friedrich-Alexander-Universität Erlangen-Nürnberg (FAU), Erlangen, Germany; 2 Bayreuth Center of Ecology and Environmental Research (BayCEER) and Department of Sport Science, University of Bayreuth, Bayreuth, Germany

**Keywords:** Ecology, Evolution, Ocean sciences

## Abstract

Crustose coralline red algae (CCA) play a key role in the consolidation of many modern tropical coral reefs. It is unclear, however, if their function as reef consolidators was equally pronounced in the geological past. Using a comprehensive database on ancient reefs, we show a strong correlation between the presence of CCA and the formation of true coral reefs throughout the last 150 Ma. We investigated if repeated breakdowns in the potential capacity of CCA to spur reef development were associated with sea level, ocean temperature, CO_2_ concentration, CCA species diversity, and/or the evolution of major herbivore groups. Model results show that the correlation between the occurrence of CCA and the development of true coral reefs increased with CCA diversity and cooler ocean temperatures while the diversification of herbivores had a transient negative effect. The evolution of novel herbivore groups compromised the interaction between CCA and true reef growth at least three times in the investigated time interval. These crises have been overcome by morphological adaptations of CCA.

## Introduction

Coral reefs support the biologically most diverse marine ecosystems and have done so over substantial parts of earth history, starting in the Late Triassic, when scleractinian corals became prolific reef builders^1^. Mitigating the threats to modern coral reef ecosystems will thus benefit from a better understanding of the underlying causes in the rise and fall of ancient coral reefs^2^. Reefs, broadly defined as laterally confined limestone “structures built by the growth or metabolic activity of sessile benthic aquatic organisms”^3^ comprise a large array of constructional styles and biota, and grew in a variety of environments. The concept of ‘true reefs’ in ancient reefs derives from the constructional style and environment of recent tropical, shallow-water coral reefs. True reefs with a syndepositional relief and a rigid framework constructed by skeletal organisms are known since Cambrian times^4^ but occur alongside other reef types such as reef mounds, mud mounds and biostromes. There is a significant increase of both the relative and absolute abundance of true reefs over the Phanerozoic^5^. This trend may be caused by intrinsic (biological) factors as there are few significant correlations with earth system parameters such as temperature, sea level, or oceans chemistry^5^. Although hypothesized to play a major role^6^, the relative importance of biotic interactions in reef evolution is still poorly known. The evolution of crustose coralline red algae (Subclass Corallinophycidae Le Gall and Saunders^7^, hereafter referred to as ‘CCA’) may underline such interactions in an exemplary fashion. CCA play a key role in the construction of many modern coral reefs^8–10^ in several regards: CCA are not only primary producers and contributors of calcareous sediment but also often act as consolidators and binders of coral reefs. The algae can form a distinct ridge providing a surf-resistant reef crest and bind loose sediment^11^. Even though there are examples of true reefs that grow successful without this CCA ridge under wave-exposure^12,13^, CCA are still considered as ‘the glue that holds coral reefs together’^9^ in many cases. Another key factor for the success of modern coral reefs are grazing organisms such as echinoids and parrot fish, because they remove fleshy algae from the reef surface^14,15^. The evolution of grazers also led to an increased feeding pressure on CCA, followed by adaptive strategies of the CCA^16^. It seems plausible that this development also impacted a potential capacity of CCA to facilitate coral reef formation in the geological past. However, this hypothesis has not been assessed quantitatively until now.

There are hints for a long-term positive interaction between corals and CCA over geological timescales^17^. However, a finer temporal and taxonomic resolution and the inclusion of environmental parameters may explain if and why a potential importance of CCA for reef development varies over time. Using data from the PaleoReefsDatabase (PARED), a comprehensive compendium of geological and paleontological data of Phanerozoic reef sites as described previously^18–20^, we evaluate the role of CCA in coral reef development at the level of geological stages from the Early Cretaceous to the Pleistocene while considering additional factors such as ocean temperature and chemistry as well as interactions with relevant groups of grazing organisms. We test three hypotheses: (1) The probability of true reef formation is correlated with the presence of CCA reef cementers; (2) the capacity of CCA to reinforce coral reefs is linked to various oceanographic and ecological parameters; (3) the diversification of grazing organisms led to transient crises in the capacity of CCA to support true reef formation. Additionally, we highlight alternative explanations for the correlation between CCA occurrences and the formation of true reefs. This includes the impact of herbivore radiation on corals rather than on CCA directly and ecological niches for CCA provided by increased reef growth.

## Results

**Figure 1:**
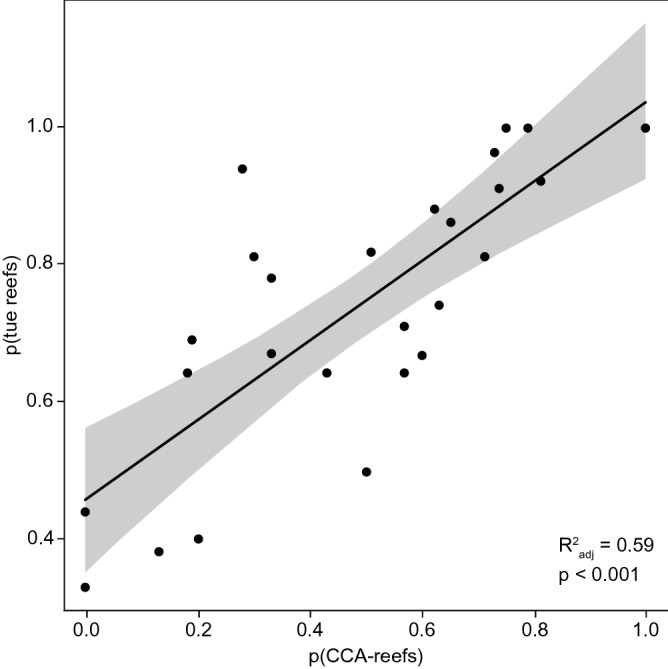
Role of CCA as secondary reef builders. Linear regression model between proportions of true reefs and proportions of coral reefs with CCA as secondary reef builders indicating a significant correlation between CCA cementation and true reef development.

The general hypothesis that the occurrence of true reefs is strongly correlated with CCA as secondary reef builders is supported by the linear regression model (Fig. 1). Regression residuals are not auto-correlated suggesting that no further data treatment is required. (Supporting Information Fig. S1).

**Figure 2:**
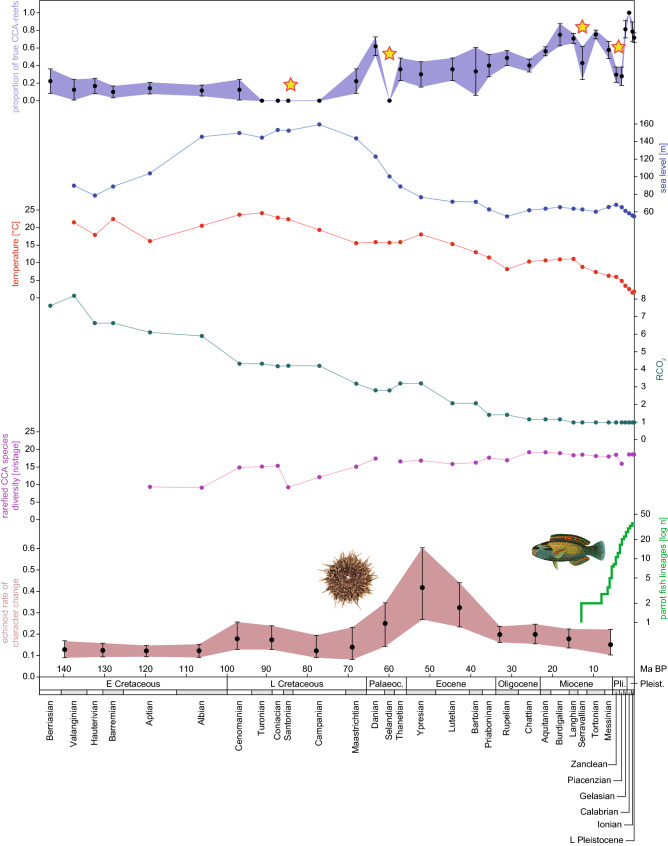
Temporal patterns of true CCA-reef formation and potentially influencing parameters. Patterns of investigated variables, representing proportions of true CCA-reefs retrieved from PARED, relative sea level based on ocean volume change, ocean temperature derived from oxygen isotope data, CO_2_ concentration relative to the current level, rarefied species diversity of CCA, echinoid evolution expressed as mean number of character changes per lineage per million years, and parrot fish origination and diversification expressed as lineage-through-time plot. Stars indicate the four transient crises in the CCA’s abundance within true coral reefs.

The temporal patterns of the investigated variables, proportions of true CCA-reefs, sea level, ocean temperature, CO_2_ concentration, species diversity of CCA, and the origin and diversification patterns of the grazer clades echinoids and parrot fish are visualized in Fig. 2. Four transient crises are evident, one between the Turonian and the Campanian, one in the Paleocene (Selandian–Thanetian), one in the Miocene (Serravallian), and the youngest one in the Pliocene (Zanclean–Piacenzian).

All environmental variables except grazers are correlated with each other (Table 1). The high values of correlation between potential explanatory variables hinder a quantification of independent effects in the subsequent analyses. The only exceptions are the origination and diversification patterns of grazers.

**Table 1:**
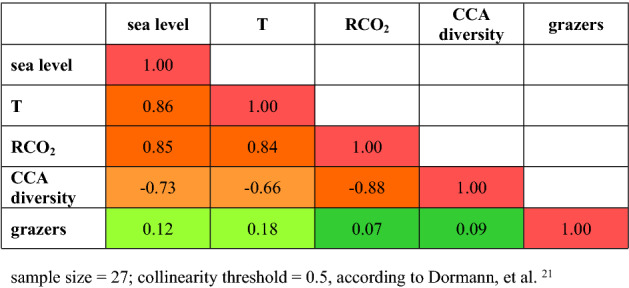
Collinearities between environmental parameters.

Sample size = 27; collinearity threshold = 0.5, according to Dormann, et al.^21^.

**Figure 3:**
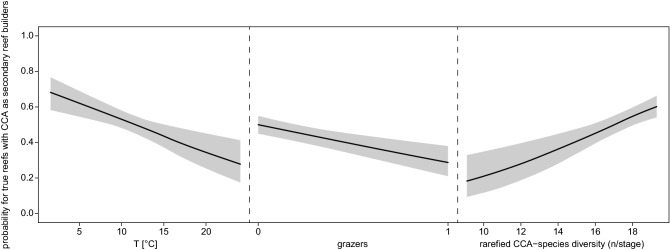
Visualization of the GLM. CCA species diversity is positively correlated with the presence of true coral reefs that have CCA as secondary reef builders while origins and higher ocean temperatures have a negative effect.

Stepwise model selection in a multivariate GLM selected the variables ocean temperature, grazers, and species diversity of CCA for the final model (Table 2) with reasonable goodness of fit measures (Table 3, Fig. S2). The evaluation of residuals shows that residuals are not significantly auto-correlated (Supporting Information Fig. S3). However, the high correlation among explanatory variables (Table 1) makes it impossible to separate the effects of CCA diversity and ocean temperature with confidence_._ Nevertheless, visualization of the GLM shows that species diversity of CCA is positively correlated with the presence of true CCA-reefs while higher ocean temperatures and the origination and diversification of grazers have negative effects (Fig. 3).

**Table 2:** Results of the GLM.

	AICc	Estimate	*p* value
- None	125.39		
- T	135.76	− 0.08 ± 0.02	< 0.001
- Grazers	138.66	− 0.88 ± 0.22	< 0.001
- CCA diversity	141.68	0.19 ± 0.05	< 0.001

Null deviance: 148.84 on 26 degrees of freedom.

Residual deviance: 43.34 on 23 degrees of freedom.

D^2^ = 0.71, D^2^_adj_ = 0.67.

**Table 3:** Goodness of fit measures for the GLM.

	Mc Fadden’s pseudo-R^2^	Maximum likelihood pseudo-R^2^	Nagelkerke’s pseudo-R^2^
GLM	0.48	0.98	0.98
T	0.30	0.92	0.92
Grazers	0.09	0.53	0.53
CCA diversity	0.35	0.94	0.94

## Discussion

### The role of CCA as reef consolidators

We found a significant correlation between the proportion of reefs that contain CCA as secondary reef builders and the proportion of true reefs over the last 150 million years. Coral reefs can benefit from CCA in various ways. Relating to the reef ridge, the stony pavement made up by the algae protects the ridge from onrushing waves and also consolidates the reef flats behind the ridges^11^. With reference to the whole reef, CCA reinforce the structure created by corals, fill cracks, bind together much of the sand, dead corals and debris, and thereby create a stable substrate and reduce reef erosion^22^. Larval settlement, metamorphosis, and recruitment of several coral species is strictly determined by chemosensory recognition of specific signal molecules uniquely available in specific CCA^23^.

However, it has to be considered that there are modern reefs that cope with wavy, high-energy environments without the aid of CCA, as for example the Alacran reef in Mexico^12^. CCA are not the only possibility to add rigidity to a reef. Submarine lithification can be more important than CCA in creating calcite precipitates, especially when environmental and ecological conditions are unfavourable for the growth of CCA, e.g. because of the lack of light. Submarine lithification in the form of Mg-calcite precipitates exists in many forms, including cemented micritic crusts and infillings of cracks. Additionally, their respective carbonate sources may be abiotic^24^ or originate from a great variety of organisms, including reef fish^25^. Therefore, they do also play an important role for the structural integrity of coral reefs^24^. CCA abundance may benefit from reef growth in terms of ecological niches provided, additionally increasing the positive correlation. We thus suggest that the significant correlation between the proportion of reefs reinforced by CCA as secondary reef builders and the proportion of true reefs can be interpreted as a mutual benefit. On the one hand, the presence of CCA can add stability to coral reefs, especially when the reef ridge is exposed to heavy wave action. On the other hand, sufficient reef growth can be a prerequisite for a larger abundance of CCA. A shift towards one side in this mutual dependence is subject to the particular features of each reef, as for example if CCA rather benefit from the shelter of crevices in reefs with high grazing pressure or if corals rather benefit from the presence of CCA at sites of intense wave exposure.

### The physicochemical parameters ocean temperature, sea level, and RCO_2_

CCA occur worldwide from the tropics^10^ to polar latitudes^26^ and temperature is one of the primary determinants in their geographical distribution, and the boundaries of their biogeographical regions are associated with isotherms^27^. Therefore, the identification of ocean temperature as an important driver of CCA reefs is reasonable. Aguirre, et al.^28^ reported that throughout the history of CCA, species richness broadly correlates with global mean palaeotemperature. However, only the diversity of the order Sporolithales varies positively with temperature, whereas the diversity of the order Corallinales varies negatively with temperature. Accordingly, the warm-water Sporolithales were most species-rich during the warm Cretaceous, but they declined and were rapidly replaced by the Corallinales as Cenozoic temperatures declined. In recent environments, members of the Sporolithales are confined to greater water depths while in euphotic reefs, they do not play a role as reef stabilizers^28^ and occupy only cryptic habitats sensu Kobluk^29^, i.e. cavities that serve as well-protected habitats and are not subject to the full spectrum of environmental and biotic controls that exist on the reef surface^28^. The wave-pounded intertidal algal ridges are built predominantly by *Porolithon onkodes* (Heydrich) Foslie 1909, *P. gardineri* (Foslie) Foslie 1909, *P. craspedium* (Foslie) Foslie 1909, and *Lithophyllum kotschyanum* Unger 1858 in the Indo-Pacific. In the Atlantic, the main reef reinforcers are *Porolithon onkodes* (Heydrich) Foslie 1909 and *Lithophyllum congestum* (Foslie) Foslie 1900. All these species belong to the ‘cool’-water adapted Corallinales. Thus, the increasing capacity of CCA to stabilize coral reefs is in line with the general trend of decreasing ocean temperatures.

A change in sea level does not impact the capacity of CCA to reinforce coral reefs, likely because sea level changes measured on the level of geological stages have no effect on reef formation^5^. On shorter time scales, sea level is expected to influence the formation of coral reefs, but probably not the CCA’s reef enforcing capacity. We conclude this because the environmental tolerances of CCA in terms of sea level fluctuation are much wider than those of reef corals. Most CCA species appear uniquely tolerant of aerial exposure^10^. Additionally, many CCA are very well adapted to changes in salinity and especially to low photon irradiances^30^. The environmental tolerances of reef corals are narrower^31,32^.

Considering our assumption that there is a mutual relationship between the presence of CCA and the growth of true reefs, another reason might be that one of the most important genera in modern coral reefs, *Acropora* Oken, 1815, is well adapted to cope with rapid sea-level changes. First observed as an important reef builder in the Oligocene^33^, *Acropora* has become a dominant reef builder from the Pleistocene until today, when sea-level fluctuations increased in rate and magnitude^34^. Indeed, there is a temporal overlap between the first decline in the fraction of CCA reefs—between the Turonian and the Campanian—and a maximum in sea level. Despite this, sea level is not selected as a relevant explanatory variable for the fraction of CCA reefs by the GLM because the relationship between the fraction of CCA reefs and sea level varies inconsistently throughout entire time series of the analysed 150 million years. While the decline in the fraction of CCA reefs may additionally be linked to an increase in temperature before and a significant drop in CCA diversity during the period with a low fraction of CCA reefs, data of the analysis are not suitable to conclusively identify the driver for this particular CCA crisis.

For the entire time series, RCO_2_, was identified by the model as a minor driver, which may be explained by the fact that an increase of atmospheric *p*CO_2_ has only little to no impact on mean ocean surface pH on timescales exceeding 10,000 years^35^. A plausible reason is that slow rates of CO_2_ release lead to a different balance of carbonate chemistry changes and a smaller seawater CaCO_3_ saturation response. This is because the alkalinity released by rock weathering on land must ultimately be balanced by the preservation and burial of CaCO_3_ in marine sediments. The burial is controlled by the CaCO_3_ saturation state of the ocean and therefore, the saturation is ultimately regulated by weathering on long time scales, and not by atmospheric *p*CO_2_. The effect of weathering on atmospheric *p*CO_2_ is much weaker than the effect of weathering on ocean pH. The much stronger effect of weathering on ocean pH allows pH and CaCO_3_ saturation to be almost decoupled for slowly increasing atmospheric *p*CO_2_^35^.

### The influence of CCA species diversity

The quantification of CCA species diversity in the geological past is associated to a number of challenges. While for recent CCA the extensive use of molecular phylogenetic methods resolved the four orders (Corallinales, Hapalidiales, Sporolithales, and Rhodogorgonales) currently recognized in the subclass Corallinophycidae as monophyletic lineages^36,37^, we have to rely on morphological characters since molecular methods are not available for the identification of fossil CCA. Because CCA show a pronounced phenotypic plasticity depending on environmental factors, their taxonomic identification depends on morphological characters like conceptacles (i.e. spore chambers) and the arrangement of cells in different areas of the thallus, features often not adequately preserved in fossil CCA. This has led to a great number of fossil CCA taxa that have been described on the basis of only a few anatomical characters of doubtful taxonomic value^38^. The inclusion of such taxa precludes fully reliable diversity estimations. To circumvent such problems, we used rarefied species data reviewed by experts on fossil CCA taxonomy^28^.

Our results show that high CCA diversity is linked to a higher abundance of CCA in true coral reefs. This might seem to contrast with the fact that in modern reefs, the wave-pounded intertidal algal ridges are built predominantly by only a few species while the ones making up the majority of diversity have a cryptic, hidden mode of life protected from full or direct exposure to major physical environmental factors and therefore do not contribute significantly to reef stabilization. However, if several CCA species were contributing to the same ecosystem function, a higher species diversity may have buffered reef systems from losing all species associated with the key function of supporting reef development^39^. As discussed in detail in the next section, the abundance of CCA in true reefs was transiently reduced four times since the Early Cretaceous. Except for the earliest crisis, this was likely caused by the origin and diversification of echinoids and parrot fish, prominent groups of bioeroding organisms that denude CCA. However, the CCA-coral reef system successfully recovered all times. We argue that this was supported by functional redundancy of CCA, because a diverse group of abundant species with a wider range of responses can help absorb disturbances^39^. This redundancy of responses to events among species within a functional group—the reef cementers—is an important component of resilience and the maintenance of ecosystem services. The amount of CCA biomass is critical in terms of the cementing capacity. Multi-species community models^40^ have shown that with consecutive native species’ extinctions at high diversity levels, species extinction usually only leads to a slight decrease in the total biomass of the native community. However, when starting from a lower initial diversity, a few consecutive species extinctions cause a relatively large biomass loss that ultimately leads to collapse. It should also be stressed that sometimes single species are responsible for the functioning of an ecosystem (i.e., keystone species), even if the ecosystem features a generally high biodiversity. Therefore, such ecosystems will decline if this key species is removed^41^.

Experiments with plants in rangelands^42^ showed that functional diversity maintains ecosystem functioning. At heavily grazed sites, some species dominant in the ungrazed communities were lost or substantially reduced. In four out of five cases, the minor species that replaced these lost ones were their functional analogues. Accordingly, we suggest that formerly less dominant but functionally analogous grazing-tolerant species increased in abundance and contributed to the maintenance of ecosystem functions. CCA species removed or reduced in biomass by grazing pressure can be replaced in terms of their ecosystem service, i.e. reef cementation, by other CCA that are better adapted to grazing.

This implies that in recent coral reef environments, areas with high CCA diversity—potentially including species occupying cryptic habitats—are more resilient against disturbance. Because the skeletal mineralogies of CCA vary considerably among species^43^, this resilience possibly applies also to future ocean acidification.

### The evolution of herbivory and transient reef crises

The data reveal four crises in the abundance of CCA within true reefs, during the Cretaceous (Turonian–Campanian), the Paleocene (Selandian–Thanetian), the Miocene (Serravallian), and the Pliocene (Zanclean–Piacenzian). The reason that the timing of the Paleocene crisis differs from the known Paleocene–Eocene crisis^20^ might be that our study focuses on the number of true reefs, while the Paleocene-Eocene crisis is expressed by a change in cumulative metazoan reef volume. Except for the first one, all crises observed here occurred synchronous with pronounced evolutionary events in clades of grazing organisms. Cementing and binding is the main function of CCA in the facilitation of true coral reefs. The decline in CCA abundance during the Selandian–Thanetian corresponds with a marked increase in the rate of morphological evolution in echinoids (Fig. 2). This includes major shifts in lifestyle and the evolution of new subclades in this group^44^, with a net trend towards improved mobility and feeding ability also on CCA^16^. Regarding the Serravallian and Zanclean–Piacenzian crises, echinoids appear to play a very minor role as their evolutionary rates constantly decreased over time^44^. However, another important clade of coralline grazers, the parrot fishes (Scarinae Rafinesque, 1810) may have become major players^45^. Although reef-grazing fish have existed for nearly 400 Ma, specialized detritivores feeding on macroalgae have only been known since the Miocene^46^. This is also in line with the radiation of acroporid corals since the mid Miocene^47^, whose branched morphologies create interstitial niches for parrot fish but also for cryptic CCA species. The parrot fishes (Scarinae) first appeared in the Serravallian^45^, which may have caused the third crisis in CCA reef cementing capacity. The lineage diversification of Scarinae was most pronounced during the Zanclean-Piacenzian, which we deem responsible for the third crisis.

The abundance of CCA in true coral reefs recovered relatively fast after all crises probably due to morphological adaptations developed within the CCA. Experiments have shown that echinoids are able to graze tissues to depths averaging 88 µm^16^, which is critical for CCA with thin crust morphologies. The resulting decline of thin crust morphologies led to the occupation of niches by branching CCA^16^. The twig-like morphologies of branching CCA prevent echinoids from denuding CCA thallus and confine this process to the tips of the branches. CCA are able to transfer nutrients within their thallus^16^. Therefore, these superficial grazing wounds can be rapidly healed if sufficient nutrient reservoirs are present in other, ungrazed parts of the algae. Meristems and conceptacles engulfed in the thallus may be another adaptation pertinent to the relatively low impact of echinoid grazing, as this is a plausible strategy to protect the reproductive and growth structures of the CCA. The more intense grazing pressure exerted by the parrot fishes, which bite CCA to an average depth of 288 µm^16^ and are able to eat the tips of branched CCA^48^ may have resulted in a greater abundance of CCA with very thick crusts. Thick-crust CCA possess larger nutrient reservoirs making them capable to recover also from grazing exerted by parrot fishes. All these adaptations and their development are congruent with the origination and diversification of the grazer clades as already outlined in other studies^16,49,50^. Today and potentially already during the geological history, CCA did not only successfully adapt to various grazer clades but even required the grazing pressure to stay free of epiphytes^49^. Here we show for the first time that the process of grazer evolution may also have affected the potential capacity of the CCA to reinforce coral reefs for three times during the geological past.

### Future implications for the capacity of CCA to reinforce coral reefs

As it concerns some of the most important biodiversity hot spots on our planet^2^, the potential future impact of the ongoing global change on the capacity of CCA to reinforce coral reefs should become a focal point of reef research. Despite the implementation of numerous mesocosm and aquaria experiments^51–53^, long-term data in the magnitude of months on CCA responses to modified environmental parameters are still sparse. Also, the change from ambient to modified parameters (e.g. *p*CO_2_, temperature) happens much faster than at natural rates.

The impact of elevated *p*CO_2_ on CCA depends on the rate of change. While fast rates are critical, slow *p*CO_2_ increase may even result in increased net calcification at moderately elevated *p*CO_2_ levels^54^. However, this comes at the cost of structural integrity of the CCA skeleton which, in turn, makes the CCA likely more susceptible to bioerosion. Bioerosion by echinoids and parrot fishes is beneficial to CCA at the present state, as it removes fast growing fleshy algae and other epiphytes^49^, but nothing is known about the future of this interaction when the integrity of the CCA skeletons is altered. Additionally, it has been shown that elevated *p*CO_2_ levels accelerate sponge reef bioerosion^55–57^. Therefore, a combination of increased bioerosion rates affecting corals and CCA might lead to strongly deteriorated conditions for coral reef formation. As outlined above, a greater CCA diversity might also increase their resilience against ocean acidification because of the great variety in skeletal mineralogies.

Regarding elevated temperatures, the outcome for CCA is unpredictable. Depending on the examined species, elevated temperatures affect CCA primary production in different ways: some species show no or negligible response^30^, some change their skeletal chemistry in terms of dolomite concentration^58^, and others respond with strongly impaired germination success^59^ or declining skeletal densities^60^. Due to the positive influence of cooler temperatures on CCA’s abundance in true reefs detected in our study, elevated temperatures will likely have a negative outcome but also here, the rate of change might be similarly important as the magnitude.

To estimate the future of CCA’s potential to facilitate coral reef growth in the face of global change, we encourage long term experiments—preferably in near-natural mesocosm studies—including the main reef stabilizing CCA species.

## Materials and methods

While CCA have existed at least since the Silurian^61^, pre-Cretaceous occurrences are scarce. We downloaded data on all reefs occurring from the earliest Cretaceous (Berriasian stage, 145.5 Ma) to the Late Pleistocene (0.01 Ma) and having corals as the main reef builders from the PaleoReefs Database (PARED, www.paleo-reefs.pal.uni-erlangen.de) in May 2018. The 736 Cretaceous to Pleistocene coral reef sites were grouped in 33 geological stages (following the International Commission on Stratigraphy^62^) representing 145 million years of Earth history with information on reef diversity and environmental parameters.

The data in PARED contain information on the constructional style of each reef, distinguishing between true reefs, reef mounds, mud mounds or banks, and biostromes. True reefs are those where skeletal organisms in growth position form a dense, rigid framework. Reef mounds share abundant skeletal organisms with no evidence for a rigid skeletal framework. Mud mounds or banks predominately consist of carbonate mud, often of microbial origin. Biostromes consist of skeletal organisms but there is no syndepositional relief^19^. We focus on coral reefs in the constructional style of true reefs, because we hypothesize that their rigidity and three-dimensionality depend on the CCA’s cementing capacity. The data in PARED also contain information on the secondary reef builders and we only asserted a significant functioning as reef cementers to the CCA when they were listed as secondary reef builders within the particular coral reef. For each geological stage, we consequently tabulated the absolute numbers of four reef types:

All reefs: all reefs in this study, regardless of their constructional style and if they have CCA as secondary reef builders.
True reefs: reefs with the constructional style of true reefs, but regardless if they have CCA as secondary reef builders or not.
CCA-reefs: reefs that have CCA as secondary reef builders, but regardless of their constructional style.
True CCA-reefs: reefs both features apply for: the constructional style of true reefs and CCA as secondary reef builders.

From these data, we calculated the proportions (mean ± one standard error) of true reefs, CCA-reefs, and true CCA-reefs for each geological stage by dividing the number of reefs of each type by the number of all reefs (Table 4). The standard error was calculated using the equation

$$SE= \sqrt{\frac{p\left(reef \,type\right)*(1-p\left(reef \,type\right))}{number \,of \,reefs}}$$

where *p(reef type)* is the calculated proportion of true reefs, CCA-reefs, and true CCA-reefs, respectively.

**Table 4:** Absolute numbers and proportions of reef types retrieved from the PaleoReefs Database (PARED).

Geological stage	Number of all reefs (n)	Number of true reefs (n)	Number of CCA-reefs (n)	Number of true CCA-reefs (n)	Proportion of true reefs (mean ± SE)	Proportion of CCA-reefs (mean ± SE)	Proportion of true CCA-reefs (mean ± SE)
Berriasian	9	7	2	2	0.78 ± 0.14	0.22 ± 0.14	0.22 ± 0.14
Valanginian	8	5	1	1	0.63 ± 0.17	0.13 ± 0.12	0.13 ± 0.12
Hauterivian	18	4	4	3	0.22 ± 0.10	0.22 ± 0.10	0.17 ± 0.09
Barremian	20	9	4	2	0.45 ± 0.11	0.20 ± 0.09	0.10 ± 0.07
Aptian	28	18	5	4	0.64 ± 0.09	0.18 ± 0.07	0.14 ± 0.07
Albian	26	18	5	3	0.69 ± 0.09	0.19 ± 0.08	0.12 ± 0.06
Cenomanian	8	3	1	1	0.38 ± 0.17	0.13 ± 0.12	0.13 ± 0.12
Turonian	9	4	0	0	0.44 ± 0.17	0.00 ± 0.00	0.00 ± 0.00
Coniacian	3	2	1	0	0.67 ± 0.27	0.33 ± 0.27	0.00 ± 0.00
Santonian	6	2	0	0	0.33 ± 0.19	0.00 ± 0.00	0.00 ± 0.00
Campanian	5	2	1	0	0.40 ± 0.22	0.20 ± 0.18	0.00 ± 0.00
Maastrichtian	9	7	3	2	0.78 ± 0.14	0.33 ± 0.16	0.22 ± 0.14
Danian	21	17	15	13	0.81 ± 0.09	0.71 ± 0.10	0.62 ± 0.11
Selandian	2	0	1	0	0.00 ± 0.00	0.50 ± 0.35	0.00 ± 0.00
Thanetian	14	9	6	5	0.64 ± 0.13	0.43 ± 0.13	0.36 ± 0.13
Ypresian	10	5	5	3	0.50 ± 0.16	0.50 ± 0.16	0.30 ± 0.14
Lutetian	14	9	8	5	0.64 ± 0.13	0.57 ± 0.13	0.36 ± 0.13
Bartonian	3	2	1	1	0.67 ± 0.27	0.33 ± 0.27	0.33 ± 0.27
Priabonian	15	10	9	6	0.67 ± 0.12	0.60 ± 0.13	0.40 ± 0.13
Rupelian	35	26	22	17	0.74 ± 0.07	0.63 ± 0.08	0.49 ± 0.08
Chattian	45	37	23	18	0.82 ± 0.06	0.51 ± 0.07	0.40 ± 0.07
Aquitanian	96	83	62	54	0.86 ± 0.03	0.65 ± 0.05	0.56 ± 0.05
Burdigalian	12	12	9	9	1.00 ± 0.00	0.75 ± 0.13	0.75 ± 0.13
Langhian	65	59	48	46	0.91 ± 0.04	0.74 ± 0.05	0.71 ± 0.06
Serravallian	7	5	4	3	0.71 ± 0.17	0.57 ± 0.19	0.43 ± 0.19
Tortonian	77	71	62	58	0.92 ± 0.03	0.81 ± 0.05	0.75 ± 0.05
Messinian	26	23	16	15	0.88 ± 0.06	0.62 ± 0.10	0.58 ± 0.10
Zanclean	27	22	8	8	0.81 ± 0.07	0.30 ± 0.09	0.30 ± 0.09
Piacenzian	18	17	5	5	0.94 ± 0.05	0.28 ± 0.11	0.28 ± 0.11
Gelasian	16	15	13	13	0.94 ± 0.06	0.81 ± 0.10	0.81 ± 0.10
Calabrian	3	3	3	3	1.00 ± 0.00	1.00 ± 0.00	1.00 ± 0.00
Ionian	14	14	11	11	1.00 ± 0.00	0.79 ± 0.11	0.79 ± 0.11
L. Pleistocene	67	64	49	48	0.96 ± 0.03	0.73 ± 0.05	0.72 ± 0.06

Our analyses aim to identify the environmental parameters, which are crucial for the capacity of CCA to facilitate the formation of true reefs. As potential explanatory variables, we explor sea level, ocean temperature, CO_2_ concentration, species diversity of CCA, and the origin and diversification patterns of the grazer clades echinoids and parrot fish. Information on sea level represents the relative sea level based on ocean volume change as mean value per geological stage^63^. Temperature data derive from the oxygen isotope dataset published by Veizer and Prokoph^64^. CO_2_ concentration for focal time periods (mean values per stage) is expressed relative to the current level (RCO_2_ multiproxy model; Berner and Kothavala^65^). Data on CCA diversity represent rarefied species diversities per stage^28^.

Data on echinoids are from Hopkins and Smith^44^ and represent rates of morphological evolution, which are measured as the mean number of character changes per lineage per million years. To quantify the diversification rates of herbivorous parrot fish, we used lineage-through-time (LTT) estimates in a dated phylogeny (Choat et al.^45^, Fig. 1). This phylogeny was obtained by analyses of three loci (16S, control region, S7I1) and comprises 16 species of the genus *Chlorurus* Swainson, 1839 (excavating feeding mode), 45 species of *Scarus* Forsskål, 1775 (scraping feeding mode), and two species of *Hipposcarus* Smith, 1956 (scraping feeding mode), all belonging to the family Labridae Cuvier, 1816. Testing the hypothesis that novel herbivore characteristics influence the capacity of CCA to facilitate the formation of true reefs, we distinguished background intervals from intervals in which distinct increases in character change or lineage origination occurred (parameter ‘grazers’ with values 1/0). All environmental parameters are compiled in Table 5.

**Table 5:** Environmental parameters.

Geological stage	Duration^a^ (Ma)	Sea level^b^ (m)	T^c^ (°C) (mean)	RCO_2_^d^ (mean)	Rarefied CCA species diversity^e^ (n/stage)	Grazer origins and diversification^f^
Berriasian	5.3000	N/A	N/A	7.60	N/A	0
Valanginian	6.3000	88.7	21.48	8.20	N/A	0
Hauterivian	3.9000	78.2	17.89	6.61	N/A	0
Barremian	5.0000	88.4	22.42	6.61	N/A	0
Aptian	13.0000	103.0	16.21	6.10	9.4	0
Albian	12.4000	144.5	20.50	5.89	9.1	0
Cenomanian	6.0000	149.0	23.66	4.32	14.9	1
Turonian	5.0000	143.9	24.09	4.32	15.2	1
Coniacian	2.8000	152.6	22.79	4.19	15.4	1
Santonian	2.3000	151.1	22.36	4.19	9.2	0
Campanian	12.9000	158.3	19.36	4.19	12.1	0
Maastrichtian	5.1000	143.1	15.64	3.20	15.2	0
Danian	4.4000	121.7	15.91	2.80	17.4	0
Selandian	2.4000	99.5	15.78	2.80	N/A	1
Thanetian	2.9000	87.8	15.85	3.18	16.6	1
Ypresian	7.2000	76.1	18.07	3.18	16.8	1
Lutetian	8.2000	70.9	15.31	2.07	15.9	0
Bartonian	3.2000	71.0	12.97	2.07	16.2	0
Priabonian	3.3000	62.2	11.54	1.42	17.7	0
Rupelian	5.5000	54.4	8.23	1.42	17.0	0
Chattian	5.3700	61.7	10.41	1.16	19.2	0
Aquitanian	2.6000	63.2	10.70	1.16	19.3	0
Burdigalian	4.4600	64.7	10.90	1.16	19.0	0
Langhian	2.1500	63.2	11.08	0.99	18.4	0
Serravallian	2.2120	62.0	8.81	0.99	18.5	1
Tortonian	4.3620	59.6	7.42	0.99	18.2	0
Messinian	1.9140	65.2	6.35	0.99	18.0	1
Zanclean	1.7320	67.2	5.99	0.99	18.5	1
Piacenzian	1.0120	64.5	4.96	0.99	16.0	1
Gelasian	0.7820	60.8	3.43	0.99	N/A	0
Calabrian	1.0250	58.2	2.65	0.99	18.6	0
Ionian	0.6550	55.5	1.63	0.99	18.6	0
L. Pleistocene	0.1143	54.2	1.86	0.99	18.6	0

^a^Cohen, K.M., Harper, D.A.T., Gibbard, P.L. 2017. ICS International Chronostratigraphic Chart 2017/02. International Commission on Stratigraphy, IUGS. www.stratigraphy.org (visited: 2017/01/03).

^b^Müller et al.^63^.

^c^Dataset by Veizer and Prokoph^64^. To adjust for the long-term trend in oxygen isotopic composition of seawater and to calculate the temperature, we followed Veizer and Prokoph^64^. We detrended the time series using the equation: δ^18^O_*pw*_(‰) = − 0.00003‰ t^2^ + 0.0046‰ t, with *pw* being Phanerozoic seawater in standard mean ocean water (SMOW) and t being age in Ma. We calculated the temperature using the equation: T (°C) = 16.9 − 4(δ^18^O − SMOW − 0.27).

^d^Berner and Kothavala^65^.

^e^Aguirre et al.^28^.

^f^Data for parrot fish based on Choat et al.^45^. Data for echinoids based on Hopkins and Smith^44^.

We performed all statistical tests in R version 3.5.2^66^ and removed all stages containing missing values in any parameters (Berriasian to Barremian, Selandian, and Gelasian) before analyses. We tested the general hypothesis that the occurrence of true reefs is strongly linked to CCA as secondary reef builders using a linear regression model between the proportions of true reefs and the proportions of CCA-reefs and tested the regression residuals for autocorrelation.

Prior to further analysis, we tested all explanatory variables for collinearity. We then assessed the influence of the environmental parameters on the coral-coralline interaction by implementing multivariate analyses (generalized linear models, GLM). As the dependent variables represent proportions (percentage of true CCA-reefs relative to all reefs), we implemented GLMs with a binomial error distribution (logit-link function). The binomial error distribution for percentage values has the advantage to account for the fact that a particular percentage value is more accurate if it is based on a larger number of observations (here the number of reefs). We used stepwise model selection based on a version of the Akaike information criterion (AIC, which is used in statistics), that has a correction for small sample sizes (AICc) to estimate the relevance of different environmental parameters. We quantified model performance using different goodness of fit measures (Nagelkerke’s pseudo-R^2^, Mc Fadden’s pseudo-R^2^, maximum likelihood pseudo-R^2^), tested model residuals for autocorrelation and visualised the model predictions.

## Supplementary information

Supplementary information.

## Data Availability

The reef data supporting the results are freely available in the PaleoReefs Database (PARED).
