# Supplementary material for: A possible link between coral reef success, crustose coralline algae and the evolution of herbivory
Source: Sci Rep. 2020 Oct 20;10:17748. doi: 10.1038/s41598-020-73900-9 (PMC7575568; doi:10.1038/s41598-020-73900-9)

Supplementary Information to

A possible link between coral reef success, crustose coralline algae and the evolution of herbivory

Sebastian Teichert[1*], Manuel Steinbauer[1,2], Wolfgang Kiessling[1]

[1]Fachgruppe Paläoumwelt, GeoZentrum Nordbayern, Friedrich-Alexander-Universität Erlangen-Nürnberg (FAU), Erlangen, Germany

[2] Bayreuth Center of Ecology and Environmental Research (BayCEER) & Department of Sport Science, University of Bayreuth, Bayreuth, Germany

**corresponding author: sebastian.teichert@fau.de*

ORCIDs

S. Teichert: 0000-0002-3047-5539

M. Steinbauer: 0000-0002-7142-9272

W. Kiessling: 0000-0002-1088-2014

Figure S1 | Autocorrelation function of the linear regression model residuals indicating no autocorrelation

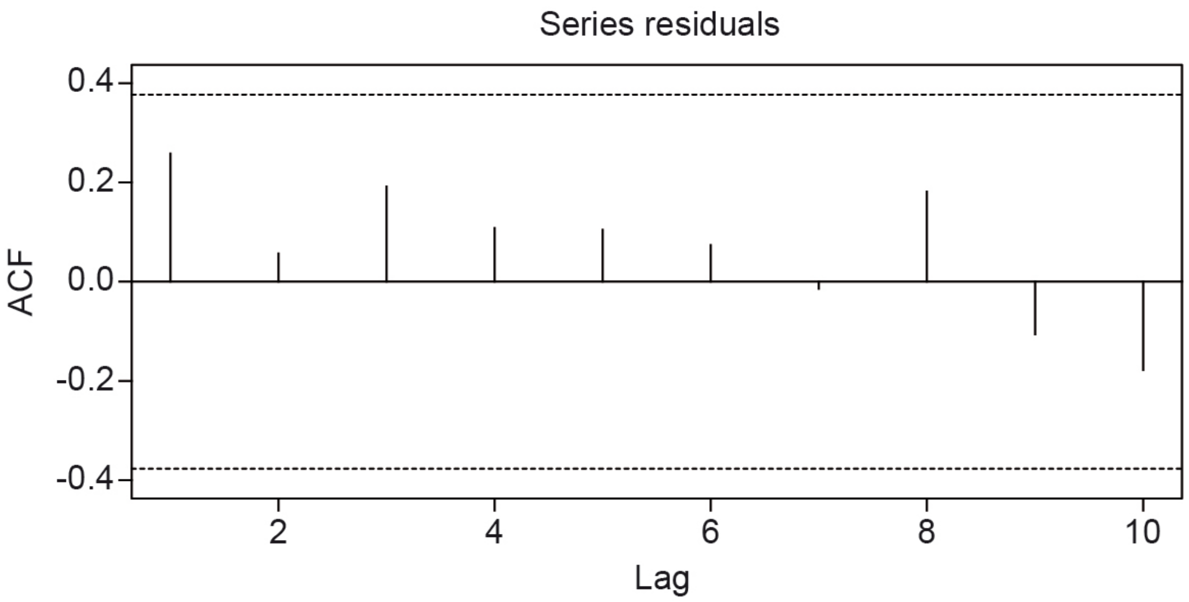

Figure S2 | Diagnostic plots of the Generalized Linear Model

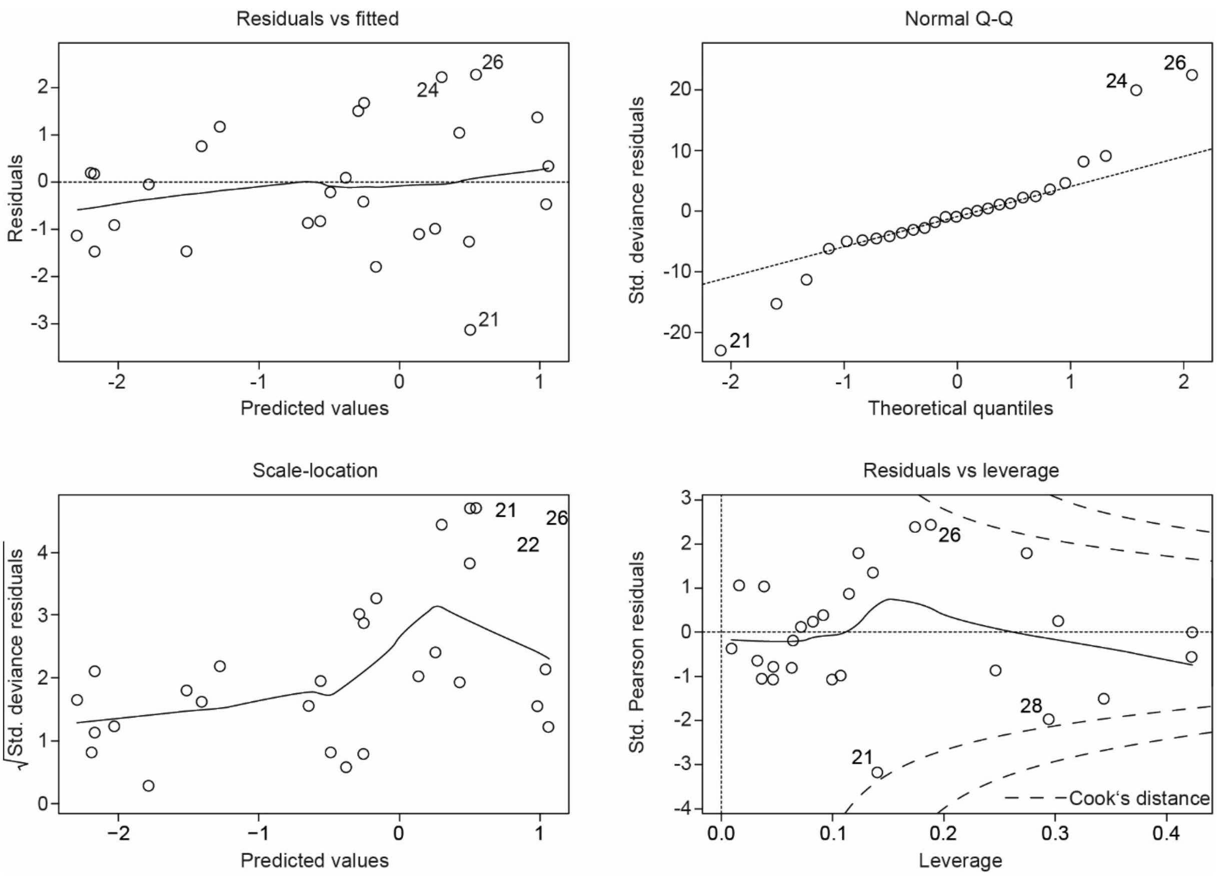

Figure S3 | Autocorrelation function of the GLM's residuals indicating no significant autocorrelation

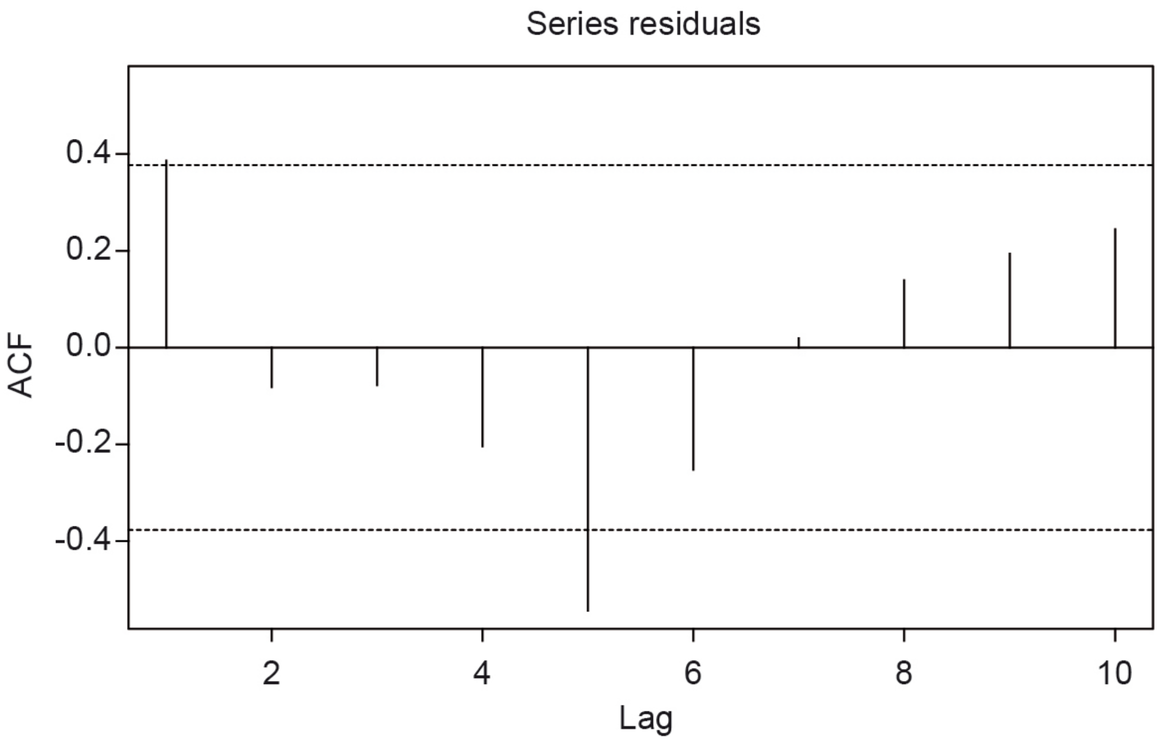

Supplement: Supplementary file 1 — Supplementary information. [file 41598_2020_73900_MOESM1_ESM.pdf]
